# Microarray Evaluation of Antimicrobial Resistance and Virulence of *Escherichia coli* Isolates from Portuguese Poultry

**DOI:** 10.3390/antibiotics5010004

**Published:** 2016-01-13

**Authors:** Nuno Mendonça, Rui Figueiredo, Catarina Mendes, Roderick M. Card, Muna F. Anjum, Gabriela Jorge da Silva

**Affiliations:** 1 Faculty of Pharmacy, University of Coimbra, Health Sciences Campus, Azinhaga de Santa Comba, 3000-458 Coimbra, Portugal; nuno.mendonca@uc.pt (N.M.); rfig86@gmail.com (R.F.); catarrinamendes@gmail.com (C.M.); 2 Centre for Neuroscience and Cell Biology, University of Coimbra, 3000-548 Coimbra, Portugal; 3 Department of Bacteriology, Animal and Plant Health Agency, Weybridge, New Haw, Addlestone, KT15 3NB Surrey, UK; Roderick.Card@apha.gsi.gov.uk (R.M.C.); Muna.Anjum@apha.gsi.gov.uk (M.F.A.)

**Keywords:** *Escherichia coli*, microarray, virulence factors, antibiotic resistance, poultry

## Abstract

The presence of antimicrobial resistance and virulence factors of 174 *Escherichia coli* strains isolated from healthy Portuguese *Gallus gallus* was evaluated. Resistance profiles were determined against 33 antimicrobials by microbroth dilution. Resistance was prevalent for tetracycline (70%) and ampicillin (63%). Extended-spectrum beta-lactamase (ESBL) phenotype was observed in 18% of the isolates. Multidrug resistance was found in 56% of isolates. A subset of 74 isolates were screened by DNA microarrays for the carriage of 88 antibiotic resistance genes and 62 virulence genes. Overall, 37 different resistance genes were detected. The most common were *tet*(A) (72%), *bla*_TEM_ (68%), and *sul1* (47%), while 21% isolates harbored an ESBL gene (*bla*_CTX-M_ group 1, group 2, or group 9). Of these, 96% carried the increased serum survival (*iss*) virulence gene, while 89% presented the enterobactin siderophore receptor protein (*iroN*), 70% the temperature-sensitive hemagglutinin (*tsh*), and 68% the long polar fimbriae (*lpfA*) virulence genes associated with extraintestinal pathogenic *E. coli*. In conclusion, prevalence of antibiotic resistant *E. coli* from the microbiota of Portuguese chickens was high, including to extended spectrum cephalosporins. The majority of isolates seems to have the potential to trigger extraintestinal human infection due to the presence of some virulence genes. However, the absence of genes specific for enteropathogenic *E. coli* reduces the risk for human intestinal infection.

## 1. Introduction

The use of antibiotics as growth promoters in animal production has led to the development of major reservoirs of antibiotic resistance genes among bacterial strains that could enter the food-chain, leading to an increased risk for human health. In 2006, the European Union limited these types of drugs to prophylactic and therapeutic use [1].

According to the Eurostat, the overall production of poultry meat for human consumption in Europe in 2012 passed 11,000 tons, among which *Gallus gallus* represents the major section. However, a recent report revealed alarming rates of antibiotic resistance among *Escherichia coli* isolated from all types of fowl production (intensive, extensive, layers, and breeding) [2]. Across Europe, *E. coli* resistance to ampicillin reached 44.1%, 40.5% to ciprofloxacin, 50.8% to sulfonamides, and 45.2% to tetracyclines [2], largely associated with several foodborne outbreaks across Europe [3].

According to the report of the European Centre for Disease Prevention and Control (ECDC), under the EARS-Net surveillance program, among human clinical *E. coli*, 57.4% of the strains were resistant to aminopenicillins, while 22.3% were resistant to fluoroquinolones [4]. Although these resistance rates are mainly associated with the consumption of antibiotics within the clinical environment, acquisition of resistance can be potentiated by dissemination of mobile genetic elements, like plasmids from bacteria present in the food chain [5]. Furthermore, it was demonstrated that, at least for some avian pathogenic *E. coli* (APEC) strains, the virulence genotyping was similar to human uropathogenic *E. coli* (UPEC), suggesting a food-borne link between APEC and UPEC [6,7].

To our knowledge, no data have been reported from Portugal regarding the resistance and virulence of *E. coli* collected from poultry. Therefore, in this study we have examined the burden of antibiotic resistance and virulence factors harbored by *E. coli* isolates recovered from healthy Portuguese chickens. Isolates (*n* = 174) were examined for susceptibility to 33 antimicrobials of therapeutic relevance in humans and poultry infections. Subsequently, a subset of isolates (*n* = 74) demonstrating resistance to at least one antibiotic was screened for the presence of 88 antimicrobial resistance genes and 62 virulence genes by using a DNA microarray.

## 2. Results

The susceptibilities of the 174 isolates were tested by Microscan, and 150 (86%) had resistance to at least one antibiotic in the panel and 24 (14%) were susceptible to all antibiotics tested. The number of isolates resistant to each antibiotic tested is presented in Table 1, and the most frequent resistances were to tetracycline (*n* = 121, 70%), ampicillin (*n* = 109, 63%), mezlocillin (*n* = 102, 59%), moxifloxacine (*n* = 98, 56%), and piperacillin (*n* = 97, 56%).

A lower frequency of resistance was detected against other antibiotics used in human therapeutics, such as trimethoprim-sulfamethoxazole (33%), gentamycin (17%), and amoxicillin plus clavulanic acid (17%). Resistance to cefotaxime was present among 21% of strains and associated with the production of ESBL. All isolates were susceptible to meropenem, ertapenem, imipenem, amikacin, and tigecycline. Multidrug resistance, defined as non-susceptibility to at least one agent in three or more antimicrobial classes [8], was detected in 56% of strains. No isolate was extensively drug-resistant or pandrug-resistant according to the criteria of Magiorakos *et al.* [8].

**Table 1:** Summary of antibiotic resistances detected in *E. coli* strains isolated from *Gallus gallus* from different types of production.

Antibiotic	Type of Poultry Production				Total (*n* = 174)
	Intensive Meat Production Farms (*n* = 121)	Extensive Meat Production Farms (*n* = 11)	Intensive Layers Farms (*n* = 17)	Intensive Breeding Farms (*n* = 25)	
Tetracycline	90 (74%)	8 (73%)	10 (59%)	13 (52%)	121 (70%)
Ampicillin	80 (66%)	7 (64%)	7 (41%)	15 (60%)	109 (63%)
Mezlocillin	77 (64%)	6 (55%)	7 (41%)	12 (48%)	102 (59%)
Piperacillin	73 (60%)	5 (45%)	7 (41%)	12 (48%)	97 (56%)
Moxifloxacin	73 (60%)	6 (55%)	9 (53%)	10 (40%)	98 (56%)
Ciprofloxacin	65 (54%)	5 (45%)	8 (47%)	8 (32%)	86 (49%)
Norfloxacin	66 (55%)	5 (45%)	6 (35%)	8 (32%)	85 (49%)
Cefazolin	43 (36%)	5 (45%)	1 (6%)	9 (36%)	58 (33%)
Trimethoprim-sulfamethoxazole	46 (38%)	5 (45%)	1 (6%)	5 (20%)	57 (33%)
Cefpodoxime	42 (35%)	5 (45%)	0 (0%)	8 (32%)	55 (32%)
Ampicillin plus sulbactam	34 (28%)	4 (36%)	2 (12%)	4 (16%)	44 (25%)
Levofloxacin	37 (31%)	3 (27%)	1 (6%)	2 (8%)	43 (25%)
Cefotaxime	26 (21%)	3 (27%)	1 (6%)	7 (28%)	37 (21%)
Cefuroxime	23 (19%)	2 (18%)	1 (6%)	5 (20%)	31 (18%)
Amoxicillin plus clavulanic acid	22 (18%)	3 (27%)	0 (0%)	4 (16%)	29 (17%)
Gentamicin	20 (17%)	0 (0%)	3 (18%)	6 (24%)	29 (17%)
Aztreonam	19 (16%)	1 (9%)	0 (0%)	6 (24%)	26 (15%)
Cefoxitin	19 (16%)	2 (18%)	0 (0%)	3 (12%)	24 (14%)
Chloramphenicol	13 (11%)	2 (18%)	2 (12%)	4 (16%)	21 (12%)
Ceftazidime	12 (10%)	0 (0%)	0 (0%)	7 (28%)	19 (11%)
Tobramycin	14 (12%)	0 (0%)	1 (6%)	3 (12%)	18 (10%)
Cefepime	9 (7%)	2 (18%)	0 (0%)	2 (8%)	13 (7%)
Colistin	7 (6%)	0 (0%)	0 (0%)	5 (20%)	12 (7%)
Nitrofurantoin	6 (5%)	0 (0%)	1 (6%)	0 (0%)	7 (4%)
Piperacillin plus tazobactam	1 (1%)	0 (0%)	0 (0%)	0 (0%)	1 (1%)
Fosfomycin	1 (1%)	0 (0%)	0 (0%)	0 (0%)	1 (1%)
Ertapenem	0 (0%)	0 (0%)	0 (0%)	0 (0%)	0 (0%)
Imipenem	0 (0%)	0 (0%)	0 (0%)	0 (0%)	0 (0%)
Meropenem	0 (0%)	0 (0%)	0 (0%)	0 (0%)	0 (0%)
Amikacin	0 (0%)	0 (0%)	0 (0%)	0 (0%)	0 (0%)
Tigecycline	0 (0%)	0 (0%)	0 (0%)	0 (0%)	0 (0%)

**Figure 1:**
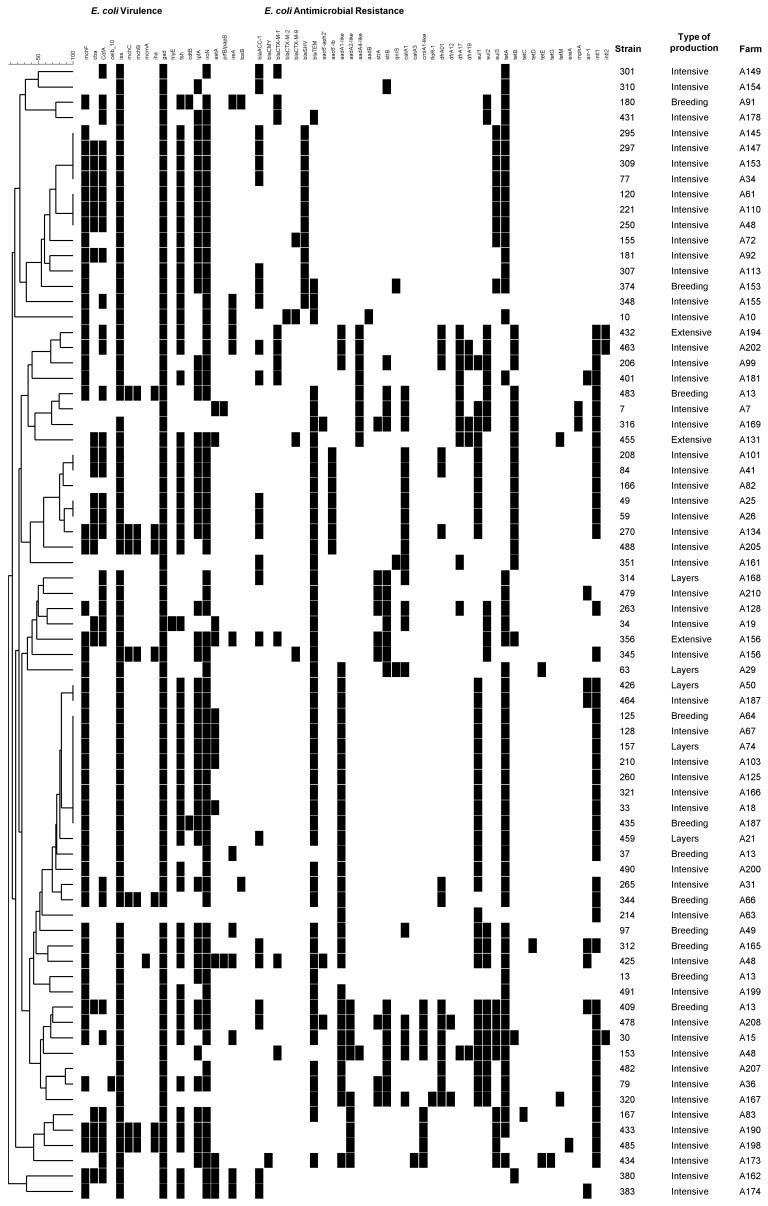
Virulence and resistance determinants detected by microarray for 74 *E. coli* isolates. The order of strains represents their relatedness according to the UPGMA dendrogram type performed in BioNumerics 5.1. The hybridization result of a distinct isolate is shown by row. A white box indicates the absence and a black box indicates the presence of the target gene.

From the total *E. coli* collection, a subset of 74 isolates presenting resistance to at least one antibiotic was selected for microarray analysis. Selected isolates were distributed over the study time and according to the proportions of the different production types. A total of 37 different resistance genes were detected in these isolates by microarray and each isolate harboured between two to fifteen genes. The most commonly detected genes were *tet*(A), *bla*_TEM_, and *sul1* (Figure 1; Table S1). In fact, 74% and 24% of the strains analyzed by microarray, with phenotypic resistance to tetracycline, presented positive signal for the *tet*(A) and *tet*(B) gene, respectively. Ten isolates were positive for a *bla*_CTX-M_ group 1, one for a *bla*_CTX-M_ group 2, and four for *bla*_CTX-M_ group 9 genes. The integrase genes *intI1* (43 isolates) and *intI2* (two isolates) were also detected, which are associated with class 1 and 2 integrons, respectively.

Virulence genes were detected by microarray in 73/74 isolates (Figure 1; Table S1). Many isolates harbored multiple virulence genes: nine isolates had from nine to eleven genes, 46 possessed six to eight genes, and 19 had five or less genes. The most commonly-detected virulence genes included: *iss*, involved in the increased serum survival (96% of isolates); *iroN*, encoding a siderophore receptor (89% of isolates); the bacteriocin gene *mchF* (72% of isolates); the temperature-sensitive hemagglutinin gene *tsh* (70% of isolates); the fimbriae encoding gene *lpfA* (68% of isolates); and the polypeptide toxin gene *cofA* (46% of isolates). The gene *gad*, encoding glutamate decarboxylase, is a housekeeping gene used as a control.

Overall, 50% of the multidrug resistance strains, for which the genotype was evaluated by microarray, presented between five to eight virulence factors and three to six antibiotic resistance encoding genes. Figure 1 also reveals that several sets of genes are observed frequently, e.g., 26% of strains presented *bla*_TEM_, *aadA1*, *sul1*, *tet*(A), and *intI1* and 8% presented *bla*_TEM_, *aac6Ib* *catA1*, *sul1*, *tet*(B), and *intI1*, all from intensive production.

## 3. Discussion

To our knowledge, in Portugal there is a lack of reports on antibiotic resistance among *E. coli* from healthy poultry and their carriage of virulence determinants. The main objective of this study was to evaluate the concomitant presence of antimicrobial resistance genes and some virulence factors among *E. coli* strains isolated from *Gallus gallus* from Portuguese farms. We detected higher resistance frequencies to several antibiotics from different classes when compared to other European countries. Resistance to ampicillin (63% *vs.* 44%), cefotaxime (21% *vs.* 6%), gentamicin (17% *vs.* 4%), and tetracycline (70% *vs.* 37%) were higher than the average resistance among the ten members states that reported to EFSA. However, these Portuguese isolates presented a similar resistance profile to that described in Spain [2]. The resistance level against nitrofurantoin was 4%, which is higher than that detected in human clinical samples in France (1.8%), USA, and Canada (1.1%) [9]. More than half of the isolates showed multidrug resistance (56%), with 8% showing co-resistance to ciprofloxacin and cefotaxime. This co-resistance decreases therapeutic options as both antibiotics are representative of major classes used in treatment of *E. coli* infections. Again, this value was higher than those reported in most European countries, with the exception of Spain where 20.8% of isolates showed this co-resistance [2]. The prevalence of resistance to aminopenicillins, third-generations cephalosporins and fluoroquinolones was higher in these poultry isolates than in Portuguese human clinical *E. coli* strains [4]. No carbapenem resistance phenotype was detected in these poultry isolates. Antimicrobial resistant food-borne *E. coli* can colonize the human gut and constitute a reservoir for subsequent infection [6]. In fact, diverse studies have associated resistant *E. coli* collected from retail meat, especially poultry, to extraintestinal human disease. Many of these zoonotic isolates belonged to B2 and D phylogenetic groups, the most commonly found in human urinary tract infections. Thus, it was suggested that poultry could be considered one of the reservoirs of antimicrobial resistant *E. coli* causing human UTIs [7]. Although B2 and D phylogroups are considered the most virulent groups, we cannot discard the association of other phylogenetic groups, such as A and B1, with UTIs [10]. In this study, the virulence microarray allowed the rapid detection and designation of pathotypes, therefore screening for specific or novel virulence combinations and the classification in phylogroups of this bacterial population was not performed.

Prevalent antimicrobial resistance genes included *tet*(A), *bla*_TEM_, and *sul1* genes, which are common in clinical and non-clinical isolates [11,12,13]. Many of the resistance genes represented on the microarray can be harboured on mobile genetic elements, such as plasmids and integrons. Thus, there is the potential for these resistance genes to be transferred to other bacteria in the microbiota of poultry or human hosts, further contributing to the dissemination of resistance. The detection of type 1 and 2 integrase genes in some isolates shows a potential for gene acquisition and transfer.

The use of antibiotics in poultry production, either prophylactically or for treatment, has been considered as a major contributor to the increased resistance frequencies detected among bacteria isolated from this type of samples. Among Portuguese poultry farms, fluoroquinolones have been detected at higher levels than the maximum residue level allowed [14], which could account for the resistance to ciprofloxacin detected in this study (49%).

Antimicrobial resistance determinants detection by microarray supported the high resistance observed to beta-lactams and tetracyclines. Similar frequencies were observed in another study on poultry in China [13], while among human UTIs the frequency of genes differs between community and clinical samples [5].

ESBLs production was detected in 21% of samples. In 2012, ECDC reported that the frequency of ESBL among clinical *E. coli* in Portugal was 13% [4], which has been attributed mainly to the dissemination of resistance mechanisms or bacterial clones. In fact, already in 2007, Mendonça *et al.* described a high incidence of *bla*_CTX-M_ from group 1 among human UTI isolates [12]. Thus, the higher prevalence of ESBLs among *Gallus gallus* strains is worrisome. Resistance to fosfomycin (in one isolate) associated with beta-lactam resistance mechanism *bla*_CTX-M_ group 2, and aminoglycoside resistance mechanism *aadB*, could potentiate co-resistance in case of co-selection [15]. Despite the ban of antibiotics in animal feed in European Union countries, our results showed that more strict policies of both prophylactic and therapeutic administration of antibiotics should be considered in Portugal.

The virulence traits detected in this study revealed an almost total presence of the increased serum survival (*iss*), a distinguishing trait of avian extraintestinal pathogenic *E. coli* (ExPEC) [16]. Other important virulence traits, prevalent in these isolates were the enterobactin siderophore receptor protein (*iroN*), the temperature-sensitive hemagglutinin (*tsh*), and the long polar fimbriae (*lpfA*) normally associated with ExPECs, including uropathogenic *E. coli* (UPEC) [17,18]. *iroN* has been directly associated with human UTI, by allowing bacterial growth even under iron-limiting conditions which are found during the infection process [19], while tsh has been more frequently detected among *E. coli* from UTI than commensal *E. coli* [20]. Finally, *lpfA* has been described as an adherence determinant among *E. coli* O157:H7 [21].

None of the genes characteristic of human intestinal pathogenic *E. coli* (STEC, ETEC, EPEC, *etc.*) strains were found, such as *eae* (intimin gene), *stx1* and *stx2* (shiga toxins), or *sta1* and *sta2* (heat-stable enterotoxins ST-Ia and ST-Ib, respectively). The gene *astA* coding for the heat-stable enterotoxin 1 was found in 13 isolates, but was the only gene of the enterotoxigenic *E. coli* (ETEC) pathogroup. These findings reduce the public health concern on acquisition intestinal pathogenic *E. coli* strains. For extraintestinal pathogenic *E. coli* (ExPEC), *iroN* was common but most isolates had no other gene from this pathogroup. Only one strain showed three of the five ExPEC genes represented in the array (*ireA*, *iroN*, *prfB/papB*) [17].

Colibacillosis is a disease in poultry caused by avian pathogenic *E. coli* (APEC). It has been difficult to identify genes that define APEC strains. They cause different types of systemic extraintestinal infections probably derived from the combinations of virulence genes acquired by the causative bacterial pathogen, and host characteristics such as age and immune status. Johnson *et al.* tried to define a set of five genes to characterize highly pathogenic APEC [16]. Nevertheless, not all of these genes are part of the microarray used [16]. More recently, some data seem to indicate that APEC are not a homogeneous group and diverse combination of genes may contribute to different clinical presentations of the infection [22]. In the virulence microarray used in this study, only four genes typically associated with APECs were screened. The *lpfA* and *tsh* genes were very common but only one isolate had the *hlyE* gene and seven the *iha*. Three isolates carried three of the four APEC genes represented in the array. So, considering the current limitations of this microarray in representation of APEC genes, and the complexity in defining clearly which virulence genes characterize APECs as a pathogroup we have limited ability to predict the health risk for the chickens in this study; although this could be resolved by expanding the microarray in the future.

Nevertheless, a strong statistical relationship was detected for the iron-regulated gene homologue adhesin (*iha*) and bacteriocins (*mchB* and *mchC*) (*r* = 1.000), present in 9% of strains. In these strains, the presence of genes encoding resistance to at least two antibiotics combined with an adherence factor [23,24], and bacteriocins, suggests an increased capacity of this *E. coli* for colonization and establishment of its niche in the urinary tract [18].

## 4. Experimental Section

### 4.1. Bacterial Isolation and Phenotypic Resistance Typing

Healthy chickens (*Gallus gallus*) (*n* = 174) originating from intensive meat production farms (121, 70%), intensive layers farms (25, 14%), intensive breeding farms (17, 10%), and extensive meat production farms (11, 6%) were collected in central Portugal between November 2010 and November 2012. *E. coli* isolates were recovered from the carcasses (58%) or internal organs (42%) of the birds at the ControlVet laboratory using standard protocols, as defined in ISO 7251:2005 [25] and identified with the API 20E System (BioMérieux, Marcy l’Étoile, France). One isolate per bird was selected for further analysis.

Isolates were tested for their susceptibility to a panel of 33 antimicrobials and combinations thereof using the microdilution broth method (Microscan Panel, Siemens, West Sacramento, CA, USA) according to the Clinical and Laboratory Standards Institute guidelines (CLSI) [26]. The antimicrobial panel comprised: ampicillin, mezlocillin, piperacillin, ampicillin plus sulbactam, amoxicillin plus clavulanate, piperacillin plus tazobactam, cephazolin, ceftazidime, ceftazidime plus clavulanate, cefotaxime, cefotaxime plus clavulanate, aztreonam, cefpodoxime, cefepime, cefuroxime, cefoxitin, ertapenem, imipenem, meropenem, norfloxacin, ciprofloxacin, moxifloxacin, levofloxacin, gentamicin, amikacin, tobramycin, tetracycline, chloramphenicol, trimethoprim plus sulfamethoxazole, colistin, nitrofurantoin, fosfomycin, and tigecyclin. All susceptibility breakpoints were interpreted according to CLSI, except moxifloxacin, colistin and tigecycline for which the European Committee on Antimicrobial Susceptibility Testing breakpoint was used [27].

### 4.2. Microarray Analyses and Data Analysis

The *E. coli* isolates were cultured overnight on Luria Bertani agar plates at 37 °C and DNA extracted by lysis as described previously [10,28]. A microarray was then used to screen each DNA sample for the presence of 88 genes encoding resistance determinants [10] and 60 genes encoding virulence factors [29]. Represented on the microarray were genes encoding resistance to beta-lactams, aminoglycosides, quinolones, phenicols, tetracycline, sulfonamides, trimethoprim, macrolide, streptogramins, and ansamycins. Among the virulence array there were genes encoding to several factors, like increased serum survival, siderophere receptors, bacteriocins, toxins, fimbria and other adhesins, among others. The DNA was labeled as described previously [10,28,29], and hybridized to the microarray using the HybPlus Kit (Alere Technologies, Jena, Germany), according to the manufacturer's instructions. Microarray analysis was performed according Figueiredo *et al.* [28]. The phylogenetic relationship between bacterial strains according to the presence or absence of resistance and virulence factors genes was evaluated using the Bionumerics software 5.1 (Applied Maths, Sint-Martens-Latem, Belgium). An unweighted pair group method with arithmetic mean (UPGMA) dendrogram was calculated by simple matching of binary coefficients on the basis of the resistance and/or virulence gene set represented on the microarray (Table S1).

### 4.3. Statistical Analysis

Pearson’s correlation was used to analyze the possible relationships among or between antimicrobial resistance phenotype, genotype, and virulence factors encoding genes. Statistical analysis was performed by the use of the statistical software SPSS (IBM SPSS Statistics for Windows, Version 22.0. Armonk, NY, USA).

## 5. Conclusions

In conclusion, *E. coli* from the microbiota of healthy Portuguese chickens might have the potential to trigger human extraintestinal infection, although the risk of acquisition of specific enteropathogenic *E. coli* strains from poultry appears to be low. The high level of resistance to some antibiotics, including extended spectrum cephalosporins, and the frequency of multidrug resistance found compared to data from other European countries is worrisome and a more effective control on the prophylactic and therapeutic use of antibiotics in avian production is recommended.

## Supplementary Files

Supplementary File 1
